# Elucidating the role of *MLL1* nsSNPs: Structural and functional alterations and their contribution to leukemia development

**DOI:** 10.1371/journal.pone.0304986

**Published:** 2024-10-15

**Authors:** Hakeemah H. Al-nakhle, Hind S. Yagoub, Rahaf Y. Alrehaili, Ola A. Shaqroon, Minna K. Khan, Ghaidaa S. Alsharif

**Affiliations:** 1 Department of Clinical Laboratory Sciences, College of Applied Medical Sciences, Taibah University, Al-Madinah Al-Monawarah, Saudi Arabia; 2 Faculty of Medical Laboratory Sciences, Omdurman Islamic University, Omdurman, Sudan; Jaypee University of Information Technology, INDIA

## Abstract

**(1) Background:**

The Mixed lineage leukemia 1 *(MLL1)* gene, located on chromosome 11q23, plays a pivotal role in histone lysine-specific methylation and is consistently associated with various types of leukemia. Non-synonymous Single Nucleotide Polymorphisms (nsSNPs) have been tied to numerous diseases, including cancers, and have become valuable cancer biomarkers. There’s a notable gap in studies probing the influence of SNPs on *MLL1* protein structure, function, and subsequent modifications;

**(2) Methods:**

We utilized an array of bioinformatics tools, including PredictSNP, InterPro, ConSurf, I-Mutant2.0, MUpro, Musitedeep, Project HOPE, RegulomeDB, Mutpred2, and both CScape and CScape Somatic, to meticulously analyze the consequences of nsSNPs in the *MLL1* gene;

**(3) Results:**

Out of 2,097 nsSNPs analyzed, 62 were determined to be significantly pathogenic by the PredictSNP tool, with ten crucial *MLL1* functional domains identified using InterPro. Additionally, 50 of these nsSNPs had high conservation scores, hinting at potential effects on protein structure and function, while 32 were found to undermine *MLL1* protein stability. Notably, four nsSNPs were deemed oncogenic, with two identified as cancer drivers. The nsSNP, D2724G, between the *MLL1* protein’s FY-rich domains, could disrupt proteolytic cleavage, altering gene expression patterns and potentially promoting cancer;

**(4) Conclusions:**

Our research provides a comprehensive assessment of nsSNPs’ impact in the *MLL1* protein structure and function and consequently on leukemia development, suggesting potential avenues for personalized treatment, early detection, improved prognosis, and a deeper understanding of hematological malignancy genesis.

## 1. Introduction

The gene mixed lineage leukemia 1(*MLL1*), also known as *KMT2A* (Lysine methyltransferase 2A) is located on chromosome 11 (11q23.3). It spans a sequence of 90,343 base pairs. It consists of 37 exons [1, 2]. The expression of *MLL1* is mainly found in the nucleus and it is widely distributed in 27 tissues. It shows high levels in tissues such as the ovary, lymph node, endometrium, thyroid, and brain [2]. Moreover, *MLL1* encodes a lysine methyltransferase (KMT) comprising 3969 amino acids. It acts as a co-activator, and plays significant roles in hematopoiesis, early developmental gene regulation, and circadian gene expression control.

Taspase 1, an endopeptidase enzyme, processes *MLL1* into *MLL C* and *MLL N* fragments. These fragments form heterodimers which regulate the transcription of genes, including HOX genes [3]. The MLL1 protein contains domains such as CXXC type zinc finger domain, extended PHD domain, and bromodomain. The Su(var)3-9, Enhancer-of-zeste and Trithorax (SET) domain within this protein possesses methyltransferase activity (mono, di tri methylation) on lysine 4 of histone 3 (H3K4 me1/2/3), which is post-transcriptional modification responsible for epigenetic transcriptional activation [4].

*MLL1* primarily controls transcription initiation and elongation through modifications within target gene promoter regions [5]. Moreover, the *MLL1* protein plays a role in regulating hematopoietic cell proliferation and differentiation, as well as modulating the gene clusters of Meis homeobox 1 (*MEIS1*) and homeobox A (*HOXA*) [6]. When these genes are not appropriately regulated, they disrupt hematopoietic development, often leading to leukemia [7].

Rearrangements involving the *MLL1* gene and its associated partners are commonly observed in different types of leukemia, including precursor B cell acute leukemias (B-ALLs), T cell acute lymphoblastic leukemias (T-ALLs), acute myeloid leukemias (AMLs), myelodysplastic syndromes (MDSs) mixed lineage (biphenotypic) leukemias (MPALs) and secondary leukemias [1]. It is worth noting that *MLL1* rearranged ALL (MLL1 r ALL) is highly prevalent, affecting more than 80% of ALL diagnoses in infants under one year old, approximately 5–6% in pediatric patients, and around 15% in adults [1, 8].

Different types of tumors have been linked to somatic mutations in the *MLL1* gene. The common changes, in *MLL1*, include mutations (3.62%) and fusions (0.13%) as well as losses (0.10%), amplifications (0.07%), and *MLL1* EP300 fusions (0.19%). Patient-derived samples commonly exhibit missense mutations (54.36%), synonymous mutations (13.61%), and nonsense mutations (7.34%) are often observed as the primary mutations. In contrast, germline *MLL1* mutations are mainly frameshift (41%) and stop mutations(29%), with a smaller percentage being missense variants(18%) [2].

Single nucleotide polymorphisms (SNPs) are the genetic variations found in the human genome occurring approximately once every 100–300 base pairs. SNPs refer to single nucleotide changes within the DNA sequence, which can potentially impact the amino acid sequence of proteins and affect their structure and function [9]. Concerns have been raised about missense synonymous SNPs(nsSNPs) as they result in amino acid changes within a protein coding sequence, potentially influencing its functionality. Evaluating the effects of nsSNPs tends to require resources and time-consuming efforts. However, by using bioinformatics tools available online, it becomes both feasible and cost-effective to analyze SNPs within a gene using computational methods. Computational genomics has played a role in studying nsSNPs associated with diseases. Several algorithms have been developed to predict the consequences of nsSNPs [9]. SNPs have been linked to diseases, including cancer. Previous research has established connections between SNPs and conditions like cancer and sporadic prostate cancer [10]. These findings have led to increased focus on the role of SNPs in diagnosing and assessing the risk of cancer. SNPs are now recognized as biomarkers for cancer.

*MLL1* inhibitors are compounds designed to target the *MLL1* protein, a crucial transcription factor and histone-H3 lysine-4 (H3K4) methyltransferase [11]. Recent research highlights inhibitors that disrupt the menin-*MLL1* interaction, exhibiting tumor-suppressive effects in specific cancers like prostate, breast, liver, and lung cancer [12]. Menin plays a pivotal role in leukemia pathogenesis, particularly in the oncogenic transformation mediated by *MLL* fusion proteins (MLL-ENL, MLL-AF4, etc.), culminating in acute leukemia [13]. Biochemical and structural analyses underscore menin’s interaction with *MLL1* and its fusion proteins, highlighting its significance in recruiting them to target genes [14]. Small molecule inhibitors like MI-2, MI-3, MI-525, and MI-503, designed to disrupt the menin-*MLL1* interaction, offer potential therapeutic avenues for *MLL*-r leukemia [15]. Efforts in developing these inhibitors have shown promise in preclinical and clinical settings, offering hope for improved leukemia treatments. Any nsSNPs within this domain could potentially disrupt the interaction between the *MLL1* domain and their inhibitors. Additionally, any nsSNPs could potentially abolish the action of *MLL1* inhibitors.

SET domain inhibitors are a class of compounds that target the SET domain, a conserved catalytic domain found in lysine methyltransferases (KMTs) [16]. The SET domain is responsible for the transfer of a methyl group from S-adenosylmethionine (SAM) to the lysine residue of histone proteins, thereby regulating gene expression and chromatin structure [4]. In recent years, there has been a growing interest in developing small molecule inhibitors that specifically target the SET domain of various KMTs, including NSD1, NSD2, NSD3, MLL, ASH1L, SETD8, and others [16]. Any nsSNPs within this domain could potentially disrupt the interaction between the SET domain and their inhibitors.

It has been demonstrated that a harmful nsSNP called Q1198P was identified in the *MLL1* gene, which was associated with acute leukemia. This particular nsSNP was believed to disrupt the function of the *MLL1* protein, leading to cell growth and division [6]. The objective of our investigation is to characterize missense nsSNPs within both coding and non-coding regions of the *MLL1* gene as listed in the dbSNP database. We also aim to identify nsSNPs that have effects on protein structure and function. In this study, we selected a range of screening methods to encompass the approaches used by various bioinformatics tools. This allowed us to classify *MLL1* nsSNPs in a consensus-based manner. After performing nsSNP screening, we conducted additional *in silico* analyses that evaluated conservation, interaction, oncogenic potential, phenotypic effects, structural considerations, and post-translational modifications (PTMs).

## 2. Materials and methods

### 2.1 Retrieval of SNP Data

**Fig 1:**
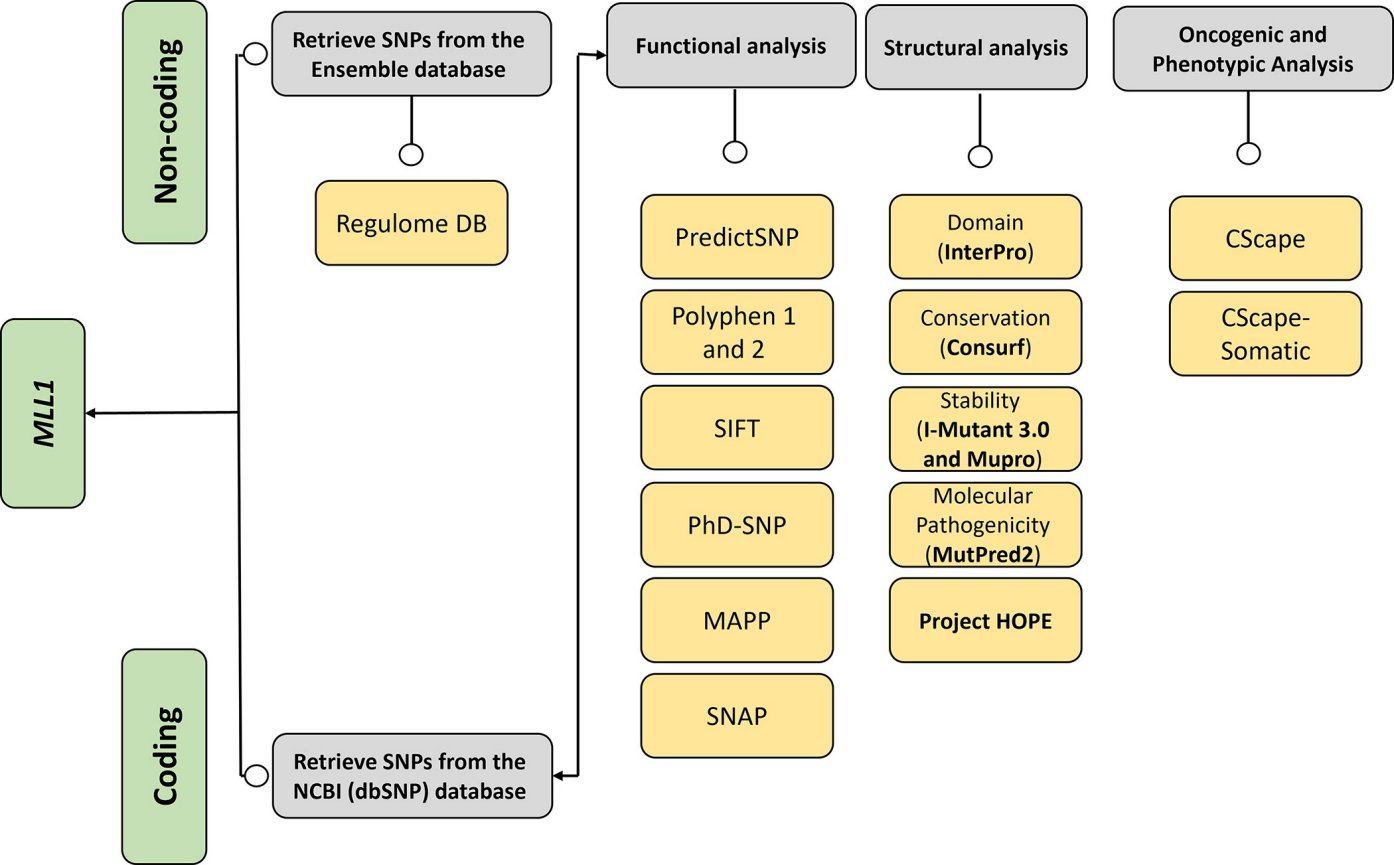
Illustrates the comprehensive methodological framework utilized in this study.

We obtained the genetic data for the human *MLL1* gene from the ENSEMBL genome browser, specifically using accession number ENST00000691053.1 [17]. To capture missense nsSNPs within both the coding and non-coding regions, we selected the transcript encoding the entire human *MLL1* protein, consisting of 3,969 amino acids. Additionally, we acquired the sequence for the *MLL1* protein from the NCBI database with accession number NP_001184033.1 (accessed on December 26, 2022) [18]. An overview of the research protocol implemented in this study is depicted in Fig 1.

### 2.2 Prediction of functional effects of nsSNPs

To assess the functional impact of nsSNPs, we utilized PredictSNP1.0 (http://loschmidt.chemi.muni.cz/predictsnp1/), accessed on December 26, 2022. PredictSNP1.0 is a consensus classifier resource that integrates predictions from six high-performing tools: SIFT, PolyPhen-1, PolyPhen-2, MAPP, PhD-SNP, and SNAP [14–19].

### 2.3 Identification of nsSNPs on MLL1 protein domains

The InterPro software located nsSNPs within conserved domains of the *MLL1* protein. InterPro (https://www.ebi.ac.uk/interpro/), accessed on December 30, 2022, identifies protein motifs and domains, aiding in functional characterization through a database of protein families, domains, and functional sites [20].

### 2.4 Analysis of protein evolutionary conservation

A ConSurf web server (https://www.ebi.ac.uk/interpro/), accessed on December 30, 2022 [21], assessed amino acid sequence conservation. This web-based algorithm estimated the degree of amino acid conservation based on multiple sequence alignment, assigning grades ranging from 1 to 9. Grade 9 indicated the most highly conserved residue, while descending numbers represented decreasing conservation levels. ConSurf also considered nucleotide and amino acid conservation and analyzed phylogenetic relationships between homologous sequences. The conserved nsSNPs in *MLL1* were further examined.

### 2.5 Analysis of nsSNP effects on protein stability

To evaluate the impact of deleterious nsSNPs on *MLL1* protein structure and stability, we utilized I-Mutant 2.0 (https://folding.biofold.org/i-mutant/i-mutant2.0.html, accessed on December 30, 2022). I-Mutant 2.0 predicted changes in protein stability by measuring the change in free energy Delta Delta G (DDG) upon mutation. A DDG value of 0 indicated reduced protein stability, while a DDG value greater than 0 signified increased stability [22].

MUpro, an additional software employed in this study, was utilized to assess the impact of nsSNPs on protein stability. This tool belongs to a category of machine learning programs that rely on support vector machines to predict the consequences of single-site amino acid substitutions on protein stability [23]. Specifically, MUpro (accessible at http://mupro.proteomics.ics.uci.edu/, accessed on December 30, 2022) utilizes the primary protein sequence of *MLL1* and employs an algorithm to forecast changes in energy, assigning a confidence score that ranges from -1 to 1. A score below 0 suggests that a single mutation is likely to reduce protein stability, while a score exceeding 0 indicates that a mutation is likely to enhance protein stability.

### 2.6 Assessing the impact of different nsSNPs on MLL1 protein structure

To analyze the impacts of nsSNPs on protein structure, we employed Project HOPE (Project Have Your Protein Explained; accessible at https://www3.cmbi.umcn.nl/hope/, accessed on February 11, 2024), an entirely automated server. The server utilizes the protein sequence in FASTA format along with SNP data as inputs. Through an amalgamation of databases and web servers including WHAT IF, UniProt, and DAS (Distributed Annotation System), Project HOPE predicts mutations. Additionally, it furnishes a homology model using YASARA for enhanced comprehension. Furthermore, Project HOPE generates a concise report elucidating how SNPs can induce alterations in protein structure and function between the wildtype and mutant forms, supplemented with pertinent annotations, images, and animations.

### 2.7 Analysis of the functional consequences of non-coding SNPs

RegulomeDB provided an annotation service for regulatory SNPs, amalgamating information derived from experimental datasets, computational forecasts, and manual annotations sourced from ENCODE. This utility assigned scores to genetic variants, enabling the differentiation of functional SNPs from a substantial pool of variants. To evaluate the impact of non-coding SNPs, the rsIDs of individual variants were submitted to the RegulomeDB database. These variants were categorized into ranks as follows: 1a-1f, indicating a high likelihood of affecting transcription factor binding and being associated with gene target expression; 2a-2c, signifying a reasonable probability of affecting binding; and 3a-3b, suggesting a lower likelihood of affecting binding. Additionally, variants classified as 4, 5, or 6 indicated minimal evidence of binding impact (S3 Table in S1 File) [24].

### 2.8 Prediction of molecular pathogenicity of nsSNPs

We employed MutPred2 (http://mutpred.mutdb.org/, accessed on December 3, 2022) to predict the structural and functional consequences of amino acid substitutions. The effects may encompass destabilizing the protein and disrupting its structure, interfering with macromolecular binding, and excising post-translational modification (PTM) sites, among others. These effects can result in significant changes in the protein’s phenotypic characteristics. Within this server, the input consisted of the FASTA sequence of the *MLL1* protein and the specific amino acid variations of interest. The P-value threshold was maintained at its default value of 0.05.

MutPred2 provided general scores (g) and property scores (p) to assess the likelihood of an amino acid substitution being deleterious or disease-associated. Missense mutations with MutPred2 (g) scores above 0.5 were considered harmful, while scores exceeding 0.75 indicated high-confidence harmful predictions [25].

### 2.9 Association of nsSNPs with cancer susceptibility

CScape and CScape-somatic were employed to forecast the oncogenic potential of the scrutinized SNPs [26, 27]. CScape-somatic boasts an impressive accuracy rate of 92% and specializes in predicting the oncogenic character of somatic point mutations within the coding regions of cancer genomes. The input format adhered to the following structure: chromosome, position, reference, and mutant, aligning with the GRCh38 assembly. The output yielded p-values, which could range from 0 to 1. A p-value exceeding 0.5 denoted a detrimental effect, while a value below 0.5 indicated benignity. Notably, CScape-somatic had the capacity to discern whether these cancer-related mutations acted as drivers or passengers. Cancer drivers typically manifest in the initial stages of tumor development, whereas passenger variants accumulate during later stages of tumor growth and typically exhibit low or negligible oncogenic potential.

## 3. Results

### 3.1 SNP annotation

We acquired *MLL1* nsSNPs from the Ensemble database, encompassing a total of 17,562 SNPs located within the intronic region, 10 SNPs in the 5′UTR region, 1,112 SNPs in the 3′UTR region, and 3,121 SNPs within the coding sequence. Among the SNPs within the coding sequence, 2,097 SNPs were of the missense type (nsSNPs), while 1,024 SNPs were synonymous. For our present study, we focused exclusively on missense nsSNPs as they induce changes in amino acids due to alterations in codons.

### 3.2 Identification of harmful nsSNPs

To identify nsSNPs with the potential to detrimentally affect the structure or function of the *MLL1* gene, we utilized PredictSNP, which integrates multiple software tools including MAPP, PhD-SNP, PolyPhen1, PolyPhen2, SIFT, and SNAP. Out of the 2,097 nsSNPs, a total of 62 were predicted to be deleterious across all computational algorithms (Table 1).

**Table 1:** Deleterious nsSNPs detected through the consensus of six in silico programs.

Domains	AA change	SNP ID	Polyphen 1 and 2	PhD-SNP, SIFT, SNAP and MAPP	Predict SNP
	C1072F	rs1085307947	Damaging	Deleterious	Deleterious
**Zinc finger, CXXC-type**	C1155Y	rs1057518074	Damaging	Deleterious	Deleterious
	C1158Y	rs1131691503	Damaging	Deleterious	Deleterious
	G1180V	rs1555038115	Damaging	Deleterious	Deleterious
	G1181C	rs1950071303	Damaging	Deleterious	Deleterious
	C1189R	rs886041875	Damaging	Deleterious	Deleterious
	C1189Y	rs1555038125	Damaging	Deleterious	Deleterious
	C1194Y	rs1950106455	Damaging	Deleterious	Deleterious
**PHD1-3**	C1448R	rs863224895	Damaging	Deleterious	Deleterious
	C1448Y C1588F	rs1085307857 rs1555042404	Damaging Damaging	Deleterious Deleterious	Deleterious Deleterious
	R1630W	rs376776245	Damaging	Deleterious	Deleterious
**Bromodomain**	W1635C	rs782594163	Damaging	Deleterious	Deleterious
	R1658W	rs373435126	Damaging	Deleterious	Deleterious
	R1763P	rs781944403	Damaging	Deleterious	Deleterious
	W1771R	rs1475344216	Damaging	Deleterious	Deleterious
	R1892C	rs1555044474	Damaging	Deleterious	Deleterious
	Y1895C	rs143373748	Damaging	Deleterious	Deleterious
	G1919R	rs1555044515	Damaging	Deleterious	Deleterious
**Extended PHD 4**	V1924M	rs1555044535	Damaging	Deleterious	Deleterious
	G1943E	rs1950444447	Damaging	Deleterious	Deleterious
	L2009W	rs1555044990	Damaging	Deleterious	Deleterious
	R2011W	rs781919638	Damaging	Deleterious	Deleterious
	G2016D	rs1397000127	Damaging	Deleterious	Deleterious
	G2027E	rs1057519403	Damaging	Deleterious	Deleterious
**F/Y-rich N-terminus**	R2067H	rs782768278	Damaging	Deleterious	Deleterious
	G2277D	rs1555046173	Damaging	Deleterious	Deleterious
	R2519W	rs782129680	Damaging	Deleterious	Deleterious
	R2521H	rs1555046685	Damaging	Deleterious	Deleterious
	D2598V	rs368088982	Damaging	Deleterious	Deleterious
	R2627C	rs1555046878	Damaging	Deleterious	Deleterious
	S2652I	rs782497028	Damaging	Deleterious	Deleterious
	R2659Q	rs1390104203	Damaging	Deleterious	Deleterious
	Y2683H	rs1555046962	Damaging	Deleterious	Deleterious
	L2700R	rs1555047001	Damaging	Deleterious	Deleterious
	**D2724G**	rs781821970	Damaging	Deleterious	Deleterious
	G2796E	rs1186580008	Damaging	Deleterious	Deleterious
	G2796V	rs1186580008	Damaging	Deleterious	Deleterious
	L2857Q	rs1057520696	Damaging	Deleterious	Deleterious
	G3000R	rs1438502727	Damaging	Deleterious	Deleterious
	S3039C	rs1279929414	Damaging	Deleterious	Deleterious
	P3098L	rs1555047613	Damaging	Deleterious	Deleterious
	G3129R	rs1353151956	Damaging	Deleterious	Deleterious
	G3186D	rs961670105	Damaging	Deleterious	Deleterious
	I3189T	rs782641177	Damaging	Deleterious	Deleterious
	S3211I	rs782372675	Damaging	Deleterious	Deleterious
	L3373H	rs781812638	Damaging	Deleterious	Deleterious
	**L3393P**	rs1555048205	Damaging	Deleterious	Deleterious
	C3430R	rs373345566	Damaging	Deleterious	Deleterious
	N3459Y	rs782596573	Damaging	Deleterious	Deleterious
	L3617P	rs146191865	Damaging	Deleterious	Deleterious
**FY-rich, C-terminal**	R3704Q	rs1555050212	Damaging	Deleterious	Deleterious
	C3743Y	rs782658611	Damaging	Deleterious	Deleterious
	**F3748C**	rs749354451	Damaging	Deleterious	Deleterious
	R3749C	rs782366377	Damaging	Deleterious	Deleterious
	R3789H	rs1555052977	Damaging	Deleterious	Deleterious
	R3810W	rs1555053010	Damaging	Deleterious	Deleterious
	R3822C	rs1591310362	Damaging	Deleterious	Deleterious
**SET domain**	**E3860D**	rs782600332	Damaging	Deleterious	Deleterious
	**G3863S**	rs1591311290	Damaging	Deleterious	Deleterious
	E3875V	rs781980190	Damaging	Deleterious	Deleterious
	R3889Q	rs1555053677	Damaging	Deleterious	Deleterious

### 3.3 Identification of nsSNPs within MLL1 domains

**Fig 2:**
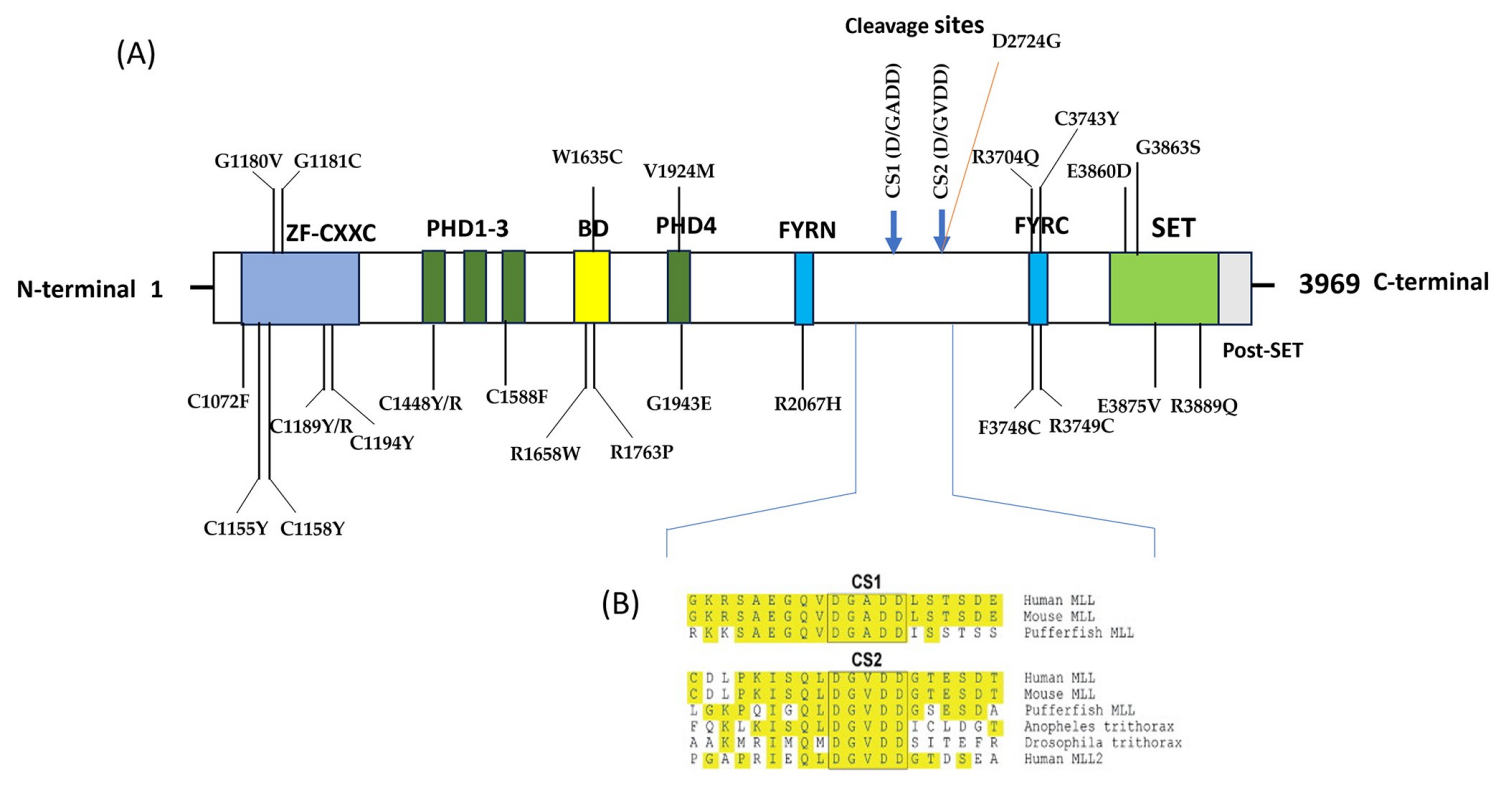
(A) Conserved domain structure of human *MLL11* with cleavage sites (CS1 and CS2). Domain and homologous superfamily of *MLL1* gene produced by InterPro. The *MLL1* protein features a CXXC domain, which consists of four cysteine residues and two zinc ions. In the N-terminal region, it possesses four plant homeotic domains (PHD), alongside a FY-rich N-terminal (FYRN) domain. Additionally, there is a FY-rich C-terminal (FYRC) domain and a catalytically active SET domain situated at the C-terminal. (B) Conservation of CS1 (D/GADD) and CS2 (D/GVDD) among species.

InterPro, a tool for domain identification, conducted a functional analysis of protein families to predict domains and active sites within the *MLL1* protein. It identified the following domains: Zinc finger, CXXC (1147–1195); Znf PHD1 finger (1433–1480); Znf PHD2 finger (1481–1531); Znf PHD3 finger (1568–1625); Bromodomain (1633–1767); Znf PHD4 finger (1932–1978); FY rich N (2021–2077); FY rich C (3666–3753); SET domain (3829–3951); and Post SET domain (3953–3969). Notably, 25 out of the 62 nsSNPs were situated within these identified domains (Fig 2, S1 Fig in S1 File and Table 1).

### 3.4 Evolutionary conservation analysis

ConSurf analysis identified that among the 62 deleterious nsSNPs found in the native *MLL1* gene, 50 were associated with highly conserved residues. The assessment of evolutionary conservation and solvent accessibility helped pinpoint structural and functional residues affected by nsSNPs in the *MLL1* gene (S2 Fig in S1 File and Table 2).

**Table 2:** Amino acid conservation scores, confidence intervals, and conservation colors produced by ConSurf.

POS	SEQ	SCORE (normalized)	COLOR	CONFIDENCE INTERVAL	CONFIDENCE INTERVAL COLOR	B/E	FUNCTION
1072	C	-0.851	9	-0.937, -0.817	9,9	b	s
1155	C	-0.851	9	-0.937, -0.817	9,9	e	f
1158	C	-0.851	9	-0.937, -0.817	9,9	b	s
1180	G	-0.861	9	-0.937, -0.842	9,9	e	f
1181	G	-0.861	9	-0.937, -0.842	9,9	e	f
1189	C	-0.851	9	-0.937, -0.817	9,9	b	s
1194	C	-0.541	8	-0.760, -0.398	9,7	b	/
1448	C	-0.856	9	-0.937, -0.817	9,9	b	s
1588	C	-0.712	9	-0.864,-0.611	9,8	b	s
1635	W	-0.799	9	-0.937,-0.760	9,9	b	s
1658	R	-0.893	9	-0.943,-0.883	9,9	e	f
1763	R	-0.635	8	-0.790,-0.565	9,8	e	f
1771	W	-0.565	8	-0.817,-0.398	9,7	b	/
1892	R	-0.893	9	-0.943,-0.883	9,9	e	f
1895	Y	-0.861	9	-0.937,-0.842	9,9	b	s
1919	G	-0.740	9	-0.883,-0.653	9,8	e	f
1924	V	-0.901	9	-0.943,-0.883	9,9	b	s
1943	G	-0.866	9	-0.937,-0.842	9,9	e	f
2009	L	-0.492	7	-0.692,-0.332	8,7	b	/
2011	R	-0.809	9	-0.900,-0.760	9,9	e	f
2016	G	-0.866	9	-0.937,-0.842	9,9	e	f
2027	G	-0.866	9	-0.937,-0.842	9,9	b	s
2067	R	-0.893	9	-0.943,-0.883	9,9	e	f
2519	R	-0.393	7	-0.611,-0.258	8,6	e	/
2521	R	-0.497	7	-0.692,-0.398	8,7	e	/
2627	R	-0.893	9	-0.943,-0.883	9,9	e	f
2652	S	-0.646	8	-0.790,-0.565	9,8	e	f
2659	R	-0.810	9	-0.900,-0.760	9,9	e	f
2683	Y	-0.728	9	-0.864,-0.653	9,8	b	s
2700	L	-0.497	7	-0.692,-0.398	8,7	b	/
2724	D	-0.893	9	-0.943,-0.883	9,9	e	f
2857	L	-0.864	9	-0.937,-0.842	9,9	b	s
3039	S	-0.842	9	-0.915,-0.817	9,9	e	f
3098	P	-0.752	9	-0.883,-0.692	9,8	e	f
3129	G	-0.313	7	-0.565,-0.175	8,6	b	/
3189	I	-0.827	9	-0.915,-0.790	9,9	b	s
3373	L	-0.610	8	-0.790,-0.514	9,8	b	/
3393	L	-0.729	9	-0.883,-0.653	9,8	e	f
3430	C	-0.269	6	-0.565,-0.083	8,5	b	/
3704	R	-0.886	9	-0.943,-0.864	9,9	e	f
3743	C	-0.846	9	-0.937,-0.817	9,9	b	s
3748	F	-0.856	9	-0.937,-0.817	9,9	b	s
3749	R	-0.698	8	-0.842,-0.611	9,8	e	f
3789	R	-0.887	9	-0.943,-0.864	9,9	e	f
3810	R	-0.887	9	-0.943,-0.864	9,9	e	f
3822	R	-0.794	9	-0.900,-0.728	9,9	b	s
3860	E	-0.882	9	-0.943,-0.864	9,9	e	f
3863	G	-0.857	9	-0.937,-0.817	9,9	b	s
3875	E	-0.882	9	-0.943,-0.864	9,9	e	f
3889	R	-0.793	9	-0.900,-0.728	9,9	b	s

Abbreviations: B, buried; E, Exposed; S, structural; F, functional; POS, position.

### 3.5 Assessment of protein structural stability

For the assessment of changes in *MLL1* stability, considering relative solvent accessibility (RI) and alterations in free energy (DDG), we employed the I-Mutant 2.0 tool, which introduced point mutations into the *MLL1* protein. The results indicated that 32 out of the 50 deleterious nsSNPs led to a decrease in stability (Table 3). Additionally, we utilized the MUpro web server to further evaluate the 32 missense substitutions that were predicted to be harmful in the previous steps (Table 3).

**Table 3:** Effect of nsSNPs on protein stability predicted by I-Mutant 2.0 and MuPro.

SNP ID	AA substitution	SVM2	RI	DDG kcal/mol	SVM3	RI	MUpro	DDG
rs1085307947	C1072F	Decrease	5	-0.49	Large decrease	1	Decrease	-0.49142
rs886041875	C1189R	Decrease	5	-1.05	Large decrease	2	Decrease	-1.01449
rs863224895	C1448R	Decrease	9	-0.72	Large decrease	2	Decrease	-1.05828
rs373435126	R1658W	Decrease	1	-0.09	Large decrease	1	Decrease	-1.51963
rs143373748	Y1895C	Decrease	6	-0.85	Large decrease	0	Decrease	-1.03143
rs1555044990	L2009W	Decrease	4	-1.32	Large decrease	1	Decrease	-1.35469
rs1397000127	G2016D	Decrease	8	-0.99	Large decrease	3	Decrease	-0.45411
rs1057519403	G2027E	Decrease	5	-1.61	Large decrease	4	Decrease	-0.50805
rs782768278	R2067H	Decrease	8	-1.39	Large decrease	7	Decrease	-1.13602
rs1555046685	R2521H	Decrease	8	-0.83	Large decrease	4	Decrease	-1.04097
rs1555046878	R2627C	Decrease	3	-1.11	Large decrease	4	Decrease	-0.73453
rs782497028	S2652I	Decrease	3	0.13	Large decrease	1	Decrease	-0.10003
rs1390104203	R2659Q	Decrease	4	-0.70	Large decrease	2	Decrease	-0.59524
rs1555047001	L2700R	Decrease	7	-0.69	Large decrease	1	Decrease	-1.82806
rs781821970	D2724G	Decrease	6	-0.89	Large decrease	6	Decrease	-1.41038
rs1186580008	G2796E	Decrease	6	-0.75	Large decrease	1	Decrease	-0.42903
rs118658000	G2796V	Decrease	6	-0.55	Large decrease	3	Decrease	-0.47996
rs1057520696	L2857Q	Decrease	9	-2.31	Large decrease	5	Decrease	-1.82752
rs1279929414	S3039C	Decrease	4	-2.02	Large decrease	6	Decrease	-0.43857
rs1555047613	P3098L	Decrease	7	-1.11	Large decrease	3	Decrease	-0.38588
rs1353151956	G3129R	Decrease	9	-1.35	Large decrease	1	Decrease	-0.28198
rs782641177	I3189T	Decrease	8	-1.31	Large decrease	5	Decrease	-2.11623
rs781812638	L3373H	Decrease	7	-1.34	Large decrease	5	Decrease	-1.74765
rs1555048205	L3393P	Decrease	3	-1.12	Large decrease	2	Decrease	-1.58189
rs782658611	C3743Y	Decrease	1	-0.23	Large decrease	3	Decrease	-1.13086
rs749354451	F3748C	Decrease	6	-1.7	Large decrease	4	Decrease	-1.82151
rs782366377	R3749C	Decrease	3	-0.85	Large decrease	2	Decrease	-1.00862
rs1555052977	R3789H	Decrease	9	-1.44	Large decrease	6	Decrease	-0.70688
rs1591310362	R3822C	Decrease	0	-0.85	Large decrease	3	Decrease	-0.95916
rs782600332	E3860D	Decrease	3	-0.39	Large decrease	1	Decrease	-1.01581
rs1591311290	G3863S	Decrease	9	-1.27	Large decrease	5	Decrease	-1.34469
rs1555053677	R3889Q	Decrease	9	-1.25	Large decrease	5	Decrease	-1.25798

Abbreviations: AA, amino acids; SVM 2 and 3, Support vector machines; RI, Reality Index; DDG, change in Gibbs free energy.

### 3.6. Investigating the impact of various nsSNPs on MLL1 3D structure

The Project HOPE server was utilized to scrutinize the consequences of single-amino acid alterations on the structure of the *MLL1* protein as showen in S4 Table in S1 File. As per the predictions obtained from this tool, mutations such as C1072F, C1155Y, C1158Y, G1180V, G1181C, C1189R, C1189Y, and C1194Y located within the Zinc finger and CXXC-type domains, as well as C1448R/Y and C1588F within PHD1-3 domain, and R1658W within the Bromo domain, and C3743Y within the FYR C-terminal domain, and E3875V within the SET domain, introduce larger substituting residues compared to the native protein, potentially leading to steric clashes. Since these point mutations occur within the mentioned domains, these clashes might disrupt the domain structure, thereby compromising its functionality. Conversely, variants such as W1635C, R1763P, R2067H, R3704Q, R3749C, D2724G, L3393P, E3860D, E3875V, and R3889Q feature smaller mutant residues relative to the size of the native residue, which could result in the loss of interactions, consequently disrupting protein functionality. Additionally, mutations introducing more hydrophobic residues at specific positions, including R1658W, R1763P, F3748C, and E3875V, might lead to the loss of hydrogen bonds and/or disrupt proper folding processes.

### 3.7 Assessment of the functional implications of non-coding SNPs

Based on the analysis conducted using the Regulome DB database, it was observed that four out of the five SNPs were assigned a ranking of 2b. This ranking suggests that a variety of data types, such as TF binding, motif information, DNase Footprint, and DNase peak, were accessible for the specific chromosomal location. Furthermore, a probability score approaching 1 strongly indicates that these particular SNPs are highly likely to be regulatory variants. Table 4 shows the results obtain from regulome DB database analysis.

**Table 4:** RegulomeDB results of the functional consequences of non-coding SNPs analysis.

Chromosome location	dbSNP IDs	Rank	Score
chr11:118522268..118522269	rs188913109	2b	0.64591
chr11:118523887..118523888	rs539251803	2b	0.6751
chr11:118524166..118524167	rs141036837	2b	0.50723
chr11:118524283..118524284	rs190548021	2b	1.0
chr11:118524539..118524540	rs147772025	3a	0.37421

### 3.8 Prediction of pathogenicity of nsSNPs

The outcome from MutPred2 analysis indicated that 32 nsSNPs were identified as deleterious, including variants such as C1072F, C1189R, C1448R, R1658W, Y1895C, L2009W, G2016D, G2027E, R2067H, R2521H, R2627C, S2652I, R2659Q, L2700R, D2724G, G2796E, G2796V, L2857Q, S3039C, P3098L, G3129R, I3189T, L3373H, L3393P, C3743Y, F3748C, R3749C, R3789H, R3822C, E3860D, G3863S, and R3889Q. The potential molecular mechanisms disrupted by these variants are summarized in Table 5, presenting the results obtained from the MutPred2 server. MutPred2 score is the average of scores from all the neutral networks of MutPred2. A score threshold of 0.05 would suggest pathogenicity.

**Table 5:** Result of MutPred2 analysis of the 32 nsSNPs, including their MutPred2 score and their impact on the different molecular mechanism.

AA variation	MutPred2 score	Molecular mechanism with P value less than 0.05
C1072F	0.926	Altered Disordered interface Altered DNA binding Gain of Strand Altered Metal binding
C1189R	0.825	Altered Disordered interface Gain of Acetylation at K1185
C1448R	0.956	Altered Metal binding Gain of Strand Altered Transmembrane protein Gain of Pyrrolidone carboxylic acid at Q1449 Loss of Sulfation at Y1447
R1658W	0.685	Loss of Intrinsic disorder Loss of Helix Gain of Loop
Y1895C	0.729	Altered Ordered interface Loss of Proteolytic cleavage at R1892 Altered Transmembrane protein
L2009W	0.662	Altered Transmembrane protein
G2016D	0.578	Altered DNA binding Altered Transmembrane protein
G2027E	0.911	Gain of Helix
R2067H	0.809	Altered Ordered interface Altered Transmembrane protein Loss of Disulfide linkage at C2068
R2521H	0.150	-
R2627C	0.782	Altered Disordered interface Loss of Intrinsic disorder Altered DNA binding Gain of Proteolytic cleavage at R2622
L2700R	0.848	Gain of Intrinsic disorder Gain of B-factor
D2724G	0.720	Altered Disordered interface Altered Metal binding Loss of Proteolytic cleavage at D2724 Gain of O-linked glycosylation at T2727
L2857Q	0.832	Gain of Intrinsic disorder Altered Disordered interface Loss of Helix
S3039C	0.249	-
P3098L	0.572	Loss of Strand Altered Transmembrane protein Gain of Pyrrolidone carboxylic acid at Q3103
G3129R	0.546	Loss of Strand Gain of ADP-ribosylation at G3129 Loss of B-factor
I3189T	0.540	Gain of O-linked glycosylation at S3185
L3373H	0.707	Gain of Intrinsic disorde
L3393P	0.740	Gain of Intrinsic disorder Altered Disordered interface
C3743Y	0.814	Altered Metal binding Gain of Loop Altered Transmembrane protein
F3748C	0.898	Altered Metal binding Loss of SUMOylation at K3752 Altered Transmembrane protein
R3749C	0.770	Loss of Intrinsic disorder Loss of SUMOylation at K3752 Altered Transmembrane protein
R3789H	0.512	Loss of Phosphorylation at Y3794 Loss of ADP-ribosylation at R3789 Gain of Sulfation at Y3794
R3822C	0.627	Altered Disordered interface Loss of Intrinsic disorder
E3860D	0.903	Gain of Allosteric site at I3859 Altered Metal binding Altered Ordered interface Gain of Relative solvent accessibility Altered DNA binding Loss of Catalytic site at E3860
G3863S	0.907	Altered Ordered interface Gain of Allosteric site at E3860 Altered Disordered interface Loss of Strand Gain of Relative solvent accessibility Altered Metal binding Altered DNA binding Gain of Catalytic site at E3860
R3889Q	0.876	Altered Metal binding Altered Transmembrane protein Altered Ordered interface Loss of Allosteric site at M3887 Gain of Relative solvent accessibility Altered DNA binding Gain of Catalytic site at R3889

### 3.9 Predicting the association of nsSNPs with cancer susceptibility

We employed CScape and CScape-somatic to assess the oncogenic potential of the screened nsSNPs. The CScape results assigned oncogenic scores of 0.650128, 0.552600, 0.766650, and 0.627944 to D2724G, L3393P, E3860D, and G3863S, respectively. Conversely, F3748C and R3789H received benign scores of 0.455280 and 0.192980, respectively, indicating a lack of association with cancer susceptibility (Table 6).

**Table 6:** Oncogenic nature mutation predicted using cscape and cscape-somatic software.

		CScape			CScape- somatic		
Variant ID	SNP	Input	Coding score	Message	Input	Coding score	Message
rs781821970	D2724G	11,118504063,A,G	0.650128	Oncogenic	11,118504063,A,G	0.552102	Driver
rs1555048205	L3393P	11,118506070,T,C	0.5526	Oncogenic (*HC)	11,118506070,T,C	0.736249	Driver
rs749354451	F3748C	11,118519714,T,G	0.45528	Oncogenic	11,118519714,T,G	0.612477	Driver
rs1555052977	R3789H	11,118520001,G,A	0.19298	Oncogenic	11,118520001,G,A	0.881861	Driver
rs782600332	E3860D	11,118521354,G,T	0.76665	Oncogenic	11,118521354,G,T	0.249088	Passenger
rs1591311290	G3863S	11,118521361,G,A	0.627944	Oncogenic	11,118521361,G,A	0.307779	Passenger

*HC = High Confidence

CScape-somatic differentiated between mutations contributing to cancer as driver or passenger variants. Driver variants are involved in tumor initiation, while passenger variants accumulate after tumor initiation and typically exhibit low or no tumorigenic potential. According to CScape-somatic, D2724G, L3393P, F3748C, and R3789H mutants were classified as driver cancer variants with scores of 0.552102, 0.736249, 0.612477, and 0.881861, respectively. In contrast, E3860D and G3863S mutants were categorized as passenger cancer variants with scores of 0.249088 and 0.307779, respectively. These classifications were based on p-value scores ranging from 0 to 1, where values above 0.5 indicated driver oncogenic potential, while values below 0.5 indicated passenger benign variants.

## 4. Discussion

The *MLL1* gene, maps into chromosome 11q23, plays an important role in the methylation of lysine residues on histones. It has been consistently linked to different types of leukemia. Moreover, nsSNPs have been associated with diseases, including cancer, and are considered valuable biomarkers for cancer. However, there is a lack of studies investigating how these nsSNPs affect protein structure, function, and modifications. This study aims to bridge that gap by identifying the nsSNPs within the *MLL1* gene and evaluating their impact on protein structure, stability, function and oncogenic potential. Additionally, this research analyzes both coding and non-coding nsSNPs associated with *MLL1* protein.

Various bioinformatics tools were employed to determine the deleterious effects caused by nsSNPs in the *MLL1* gene. Initially, the nsSNPs obtained from the dbSNP database underwent screening based on their predicted functional significance. The PredictSNP tool was used to identify nsSNPs capable of causing significant changes in the structure or function of the *MLL1* gene. Through this analysis, a total of 62 high-risk candidate nsSNPs were identified from a pool of 2049 nsSNPs.

The InterPro tool was utilized to determine the locations of the nsSNPs within the various domains of the *MLL1* protein. We found that out of the 62 identified nsSNPs, 25 are located within specific protein domains. Surprisingly, these nsSNPs are spread across ten protein domains. This distribution implies that the presence of nsSNPs in these domains could potentially disrupt their functions and activities. It is crucial to note that each domain is associated with unique functions. Consequently, the occurrence of nsSNPs in these regions can potentially affect their integrity and subsequent functional behaviors, resulting in biological consequences.

One of these domains is the CXXC domain or the zinc finger (ZF) CXXC domain. It binds and recognizes non-methylated CpG DNA sequences of target genes by coordinating two zinc ions through four cysteine residues (Cys4) [28]. The presence of these nsSNPs within this domain can potentially compromise its functionality. Moreover, maintaining the integrity of the CXXC domain protein relies on the availability of zinc ions; any mutations or nsSNPs affecting cysteine residues involved in zinc coordination may lead to the unfolding of the protein [29]. Apart, from the ZF CXXC domain, another important component of the *MLL1* protein consists of four PHD fingers referred to as PHD1 to PHD4. Each finger contains a pattern involving Cys4 His Cys3, which is controlled by two zinc ions and has a role in binding to methylated histone. This particular domain is important in preventing the development of leukemia. The incorporation of the PHD2-PHD3 finger in the chimeric MLL1-AF9 has been demonstrated to inhibit the transformation of mouse bone marrow, foster the differentiation of hematopoietic cells, and diminish Hoxa9 expression [30, 31]. Consequently, nsSNPs can negatively affect this domain’s functionality and may facilitate leukemia progression.

Between PHD3 and PHD4 lies a bromodomain (BRD), which regulates gene transcription by interacting with acetyl lysine proteins [32]. It is common for proteins with BRDs or chromatin loci, such as enhancers that recruit BRD proteins, to be disrupted in cancer cases. This disruption leads to the expression of oncogenes. The recent introduction of BRD inhibitors, including those targeting Bromo molecules, has proven effective in murine cancer models. This offers a foundation for drug development [33]. In this situation, it seems reasonable to suggest that the identified variations in the nsSNP, W1635C, R1658W, and R1763P within this domain could potentially contribute to developing blood cancer and resistance to treatments. After examining the BRD (binding domain), it becomes apparent that the protein structure contains a PHD (plant homeodomain) along with an FY-rich N-terminal domain (FYRN) and an FY-rich C- terminal domain (FYRC). These specific domains play a role in the covalent dimerization process of the proteins N and C terminal fragments following proteolytic cleavage. It is conceivable that any alterations or mutations within these domains may disrupt this dimerization process, potentially compromising the function or stability of the protein.

Lastly, we have identified another domain known as the SET domain. This particular domain is responsible for histone mono-methylation, di-methylation, or tri-methylation [34]. Any changes in its conformation caused by cancer-related nsSNPs in *MLL1* protein can lead to increased methyltransferase activity and a diminished reliance on partners. Mutant enzymes likely lose control over their activities, resulting in alterations to *MLL1* functions [35]. Variations (nsSNPs) in the SET domain have been observed in cancers, suggesting that they may have a widespread impact on gene regulation of chromatin modifications [36]. Therefore, nsSNPs located within this domain could be highly significant in cancer development and may involve in SET domain inhibitors resistance.

Using ConSurf, we identified 50 out of 62 nsSNPs as harmful due to their conservation values. Among these, 24 are functional (exposed), and 17 are structural (buried). These conserved nsSNPs can potentially affect processes such as protein-protein interactions and protein stability. It is known that alterations in buried residues at the core of proteins primarily influence their function [37].

Various types of interactions, including hydrophobic interactions, electrostatic interactions, and hydrogen bonding, play a role in determining protein stability and folding. The stability state of a protein is a factor that determines its functionality. I-Mutant and MuPro have indicated that 32 out of the identified high-risk nsSNPs could decrease protein stability. The implications of reduced protein stability are becoming more evident, often resulting in levels of protein degradation, misfolding, and aggregation [38]. Additionally, certain nsSNPs can lead to incorrect protein folding or decreased stability [39]. There is a growing belief that 25% of nsSNPs found in human populations could affect protein function by altering protein stability [40].

In this study, we utilized the MutPred2 tool to refine predictions and better understand the molecular mechanisms underlying the potential pathogenicity of variations in amino acids or nsSNPs. Interestingly, we identified eight SNPs (C1072F, C1448R D2724G, C3743Y, F3748C E3860D, G3863S and R3889Q) that modify metal binding properties. This finding is significant because of these specific positions will give the protein’s lack of metal-binding properties. We also observed that substitutions R1658W and C3743Y influenced the transmembrane function of the protein by inducing changes in its loop structure. These structural loops have the potential to impact the functionality and transmembrane properties of proteins [41, 42].

Additionally, we discovered five nsSNPs that resulted in changes to PTM sites in the MLL1 protein. These mutations included the loss of SUMOylation at K3752, the gain of Sulfation at Y3794, the gain of O-linked glycosylation at T2727, the loss of Sulfation at Y1447, and Acetylation at K1185. Overall, these nsSNPs affected three PTM sites. From these findings, it can be inferred that these harmful nsSNPs have the potential to impact the structure and functionality of MLL1 significantly.

A structural alteration analysis generated by Project HOPE corroborates this likelihood. Owing to their positioning within a profoundly conserved region, alterations in the dimensions of amino acids could impede precise protein folding, thereby interfering with protein functionality. The Project HOPE server was utilized to scrutinize the consequences of single-amino acid alterations on the structure of the MLL1 protein. As per the predictions obtained from this tool, mutations such as C1072F, C1155Y, C1158Y, G1180V, G1181C, C1189R, C1189Y, and C1194Y located within the Zinc finger and CXXC-type domains, as well as C1448R/Y and C1588F within PHD1-3 domain, and R1658W within the Bromo domain, and C3743Y within the FYR C-terminal domain, and E3875V within the SET domain, introduce larger substituting residues compared to the native protein, potentially leading to steric clashes. Since these point mutations occur within the mentioned domains, these clashes might disrupt the domain structure, thereby compromising its functionality. Conversely, variants such as W1635C, R1763P, R2067H, R3704Q, R3749C, D2724G, L3393P, E3860D, E3875V, and R3889Q feature smaller mutant residues relative to the size of the native residue, which could result in the loss of interactions, consequently disrupting protein functionality. Additionally, mutations introducing more hydrophobic residues at specific positions, including R1658W, R1763P, F3748C, and E3875V, might lead to the loss of hydrogen bonds and/or disrupt proper folding processes.

According to STRING analysis, *MLL1* interacts with ten proteins in a network that plays a role in hematopoietic cell development. The presence of an nsSNP could disrupt *MLL1* expression. Potentially contribute to malignancies.

Regulome DB provides insights into DNA properties. It helps identify regulatory elements within the human genome. Among the non-coding nsSNPs identified, rs188913109, rs539251803, rs141036837, rs190548021, and rs147772025 are predicted to have regulatory control over *MLL1* protein expression. This prediction is based on their association with transcription binding sites (matched or unmatched motifs) as having a DNase footprint accompanied by a DNase peak. A theory suggests that these specific genetic variations (nsSNPs) could impact the development of diseases by causing changes in gene expression binding of transcription factors, chromatin, and DNA methylation modification. These changes can potentially disrupt the functioning and biochemical regulation of our makeup.

This study utilized CScape and CScape somatic to evaluate whether the identified SNPs have oncogenic properties. All four nsSNPs that were identified, including L3393P, were predicted to possess characteristics associated with cancer growth. Among them, L3393P being identified with high confidence. The use of CScape somatic played a role in distinguishing between nsSNPs that can drive cancer and those that are likely to be passenger variants. Notably, the oncogenic nsSNPs, D2724G and L3393P were classified as cancer drivers, while E3860D and G3863S were passenger variants. Cancer driver mutations typically emerge in tumor development, while passenger variants tend to accumulate as the tumor progresses but exhibit lower levels of cancer-promoting activity [43].

The oncogenic nsSNP, D2724G, located between the FY N and FY C domains, is particularly intriguing. The information provided by Mutpred2 suggests that the nsSNPs lead to a loss of cleavage at D2724. This could potentially disrupt the cleavage process of the MLL1 protein. This specific cleavage is crucial for forming two fragments N p320 (N320) and C terminal p180 (C180). If this cleavage is hindered, it might affect the formation and stability of the complex formed by these fragments. This complex is significant as it is localized to a subnuclear region.

Moreover, the aspartase 1 enzyme plays a role in post-translationally cleaving *MLL1* at specific points to produce two essential subunits, p320 and p180. Any obstruction in this process can potentially disrupt the formation of a multiprotein structure. We know this structure plays a role in regulating the activity of various genes, particularly *HOX* genes. Therefore, any deviation from this process caused by the D2724G nsSNPs can affect gene expression patterns and alter cellular behavior. Considering its nature, this nsSNPs may create an environment for cancer development or progression. This raises essential possibilities that these new variations could impact the development of the genesis of hematological malignancies, potentially affecting how these disorders progress and evolve.

### Study limitations

It’s important to note that this study has limitations. It heavily relies on bioinformatic analyses to predict how nsSNPs affect protein structure and function. However, these predictions may not accurately reflect the actual biological context. It may have limitations in terms of precision. Therefore, future investigations must confirm the findings through validation. Performing research using cell cultures, animal models, or actual patient samples can provide reliable and robust findings. While this study provides knowledge about the impact of nsSNPs in the MLL1 gene on leukemia, addressing the limitations above could significantly strengthen the study’s validity and make it more applicable in a clinical setting.

When conducting computational analysis of nsSNPs, several limitations and biases must be considered. Predictive algorithms vary in their predictions due to different underlying algorithms and datasets, requiring cautious interpretation and experimental validation. Training data bias may favor well-studied variants, potentially overlooking rare or novel ones. Computational tools simplify relationships between genetic variation and protein function, but actual impacts depend on various factors. Functional prediction algorithms rely on databases with incomplete or outdated information, leading to incomplete or inaccurate predictions. Population-specific effects influence nsSNP consequences across different populations. Protein function complexity poses challenges in accurately predicting nsSNP impact. While computational predictions offer insights, experimental validation is essential to confirm nsSNP consequences and their relevance in disease. To address these limitations, multiple computational tools should be used, and efforts to improve prediction algorithms and expand functional annotation databases are crucial.

### Future perspectives

Understanding the structural and functional consequences of genetic variants is crucial in oncology, particularly in leukemia development, for several reasons. Firstly, it aids in identifying driver mutations responsible for leukemia initiation and progression. Secondly, it facilitates personalized medicine by tailoring treatments to individual genetic profiles. Thirdly, it informs drug development by identifying potential targets. Additionally, it helps predict treatment response and prognosis, guiding treatment decisions. Furthermore, it enhances understanding of disease mechanisms and uncovers new therapeutic targets. Finally, it assists in risk assessment and prevention strategies. Overall, these understanding advances leukemia research, improves treatment outcomes, and enhances patient care and survival rates.

## 5. Conclusions

This study presents a comprehensive analysis of the effects of various nsSNPs within the *MLL1* gene, a critical player in several leukemia types. Employing a myriad of analytical tools, the research identified several high-risk nsSNPs and assessed their impact on protein structure, stability, and function, thereby addressing gaps in previous *in silico* studies. The distribution of these nsSNPs across essential domains of the *MLL1* protein indicates their potential to interfere with the protein’s multifaceted functions, thereby contributing to leukemia progression.

In summary, this research provides valuable insights into the multifarious implications of nsSNPs within the *MLL1* gene, paving the way for a deeper understanding of their role in leukemia and offering a foundation for future studies and therapeutic developments. The identification of potential cancer driver mutations in the *MLL1* protein underscore the necessity for further investigations into their mechanistic contributions to disease and their potential as targets for novel therapeutic strategies.

## Supporting information

S1 File (DOCX)
