# Supplementary material for: Elucidating the role of MLL1 nsSNPs: Structural and functional alterations and their contribution to leukemia development
Source: PLoS One. 2024 Oct 15;19(10):e0304986. doi: 10.1371/journal.pone.0304986 (PMC11478856; doi:10.1371/journal.pone.0304986)
Supplement: S1 File — (DOCX) [file pone.0304986.s001.docx]

**Comprehensive In Silico Assessment of nsSNPs' Effect in the *MLL1* Protein Structure and Function: Implications for Leukemia Development**

**
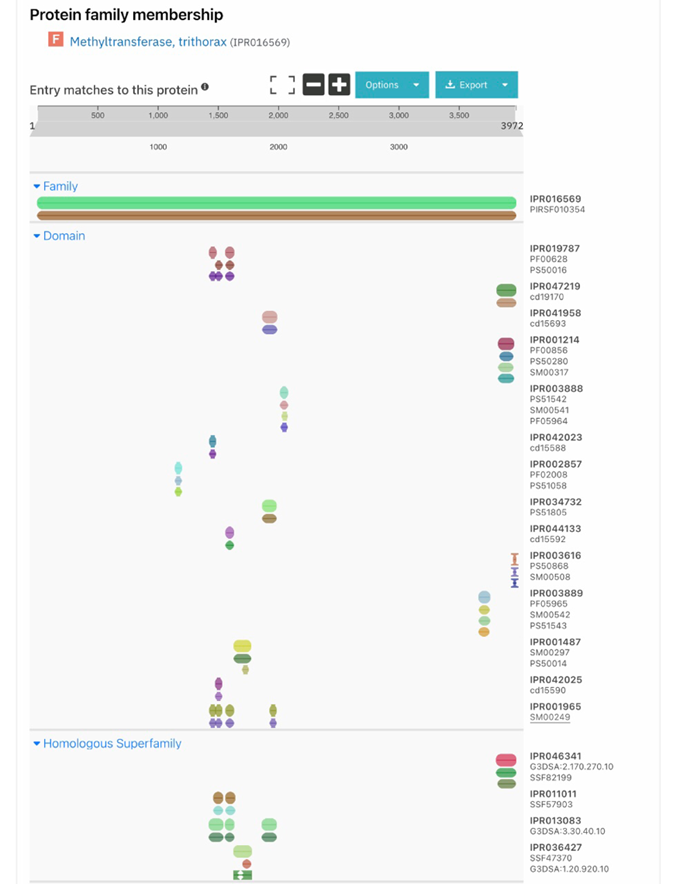
Hakeemah H Al-nakhle ^1, *^, Hind S Yagoub ^1^,^2^, Rahaf Y Alrehaili ^1^, Ola A Shaqroon ^1^, Minna K Khan ^1^, Ghaidaa S Alsharif ^1^**

**Figure S1**. Domain identification of *MLL1* protein using InterPRO server. IPR016569 indicates the *MLL1* protein (1-3969 aa), the Post SET domain (3953-3969); SET domain (3829-3951); FY rich C (3666-3753); Znf PHD4 finger (1932-1978); Bromodomain (1633-17767); Znf PHD3 finger (1568-1625); Znf PHD2 finger (1481-1531); Znf PHD1 finger (1433-1480) and Zinc finger, CXXC (1147-1195).

| 1          11         21         31         41          **MAHSCRWRFP ARPGTTGGGG GGGRRGLGGA PRQRVPALLL PPGPPVGGGG** **eeeebebebe eeeeeeeeee eeeeebeeee eeeebeebbb beeeeeeeee**   **ff**     **f f** **ff**                     **f**  **f** |
| --- |
| 51         61         71         81         91          **PGAPPSPPAV AAAAAAAGSS GAGVPGGAAA ASAASSSSAS SSSSSSSSAS** **eeeeeeeebb bbbbbeeeee eeeeeebbbb bbbbeeeeee eeeeeeeeee** |
| 101        111        121        131        141         **SGPALLRVGP GFDAALQVSA AIGTNLRRFR AVFGESGGGG GSGEDEQFLG** **ebebbbebbe ebebbbebbb bbbebbeebe ebbbebeeee eeeeeeebbe**              **fsss** **s**           **s**                    **s** **f** |
| 151        161        171        181        191         **FGSDEEVRVR SPTRSPSVKT SPRKPRGRPR SGSDRNSAIL SDPSVFSPLN** **bbeeeebebe eeeeeeeeee eeeeeeeeee eeeeebeebb eeeeebeeee**   **ff**                    **ffffffff** |
| 201        211        221        231        241         **KSETKSGDKI KKKDSKSIEK KRGRPPTFPG VKIKITHGKD ISELPKGNKE** **eeeeeeeeeb eeeeeeeeee eeeeeeebee bebebeeeee eeeeeeeeee**         **f**          **ff** **fffff**      **f**      **f**       **f** |
| 251        261        271        281        291         **DSLKKIKRTP SATFQQATKI KKLRAGKLSP LKSKFKTGKL QIGRKGVQIV** **eebeebeeee eeebeebeeb eebeeeeeee beeeeeeeeb ebeeebbebb**    **f**   **f** **f f**   **f**   **fs ff** **f**  **ffff** **ff**  **f**  **f**  **f** **f** |
| 301        311        321        331        341         **RRRGRPPSTE RIKTPSGLLI NSELEKPQKV RKDKEGTPPL TKEDKTVVRQ** **eeeeeeeeee ebeebeebbb eeeeeeeeee eeeeeeeeee eeeeeeebee** **ffffffff** **f f** **f**             **f** **f**   **f**              **f**   **ff** |
| 351        361        371        381        391         **SPRRIKPVRI IPSSKRTDAT IAKQLLQRAK KGAQKKIEKE AAQLQGRKVK** **eeeebeebeb beeeeeeebe bbeebbeebe eeeeeebeee bbeeeeeeee** **ffff** **ffsf**   **f**  **ffff**   **ssffssf** **s**  **ffffff** **fff ssffff** **f** |
| 401        411        421        431        441         **TQVKNIRQFI MPVVSAISSR IIKTPRRFIE DEDYDPPIKI ARLESTPNSR** **eebeebeebb bbbbbbbbbe bbebeeebbe eeeeeeebeb bebeeeeebe** **ffsffsffss ssssss** **ssf ssfsfffssf ffff** **ff** **f**   **f** **f** **f**   **f** |
| 451        461        471        481        491         **FSAPSCGSSE KSSAASQHSS QMSSDSSRSS SPSVDTSTDS QASEEIQVLP** **bebbebeeee eeeebeeebe ebeeeeeeee eeeeeebeee ebbeebeebe**     **fs** **fff ffff** **fffsf f** **ffffffff fff** **ffs** **ff fssf** |
| 501        511        521        531        541         **EERSDTPEVH PPLPISQSPE NESNDRRSRR YSVSERSFGS RTTKKLSTLQ** **eeeeeeeeee eebeeeeeee eeeeeeeeee bebbeeeeee ebbeebeeee**                               **ff** **f**            **ff** |
| 551        561        571        581        591         **SAPQQQTSSS PPPPLLTPPP PLQPASSISD HTPWLMPPTI PLASPFLPAS** **eeeeeeeeee eeeeeeeeee eeeeeeebee ebebbbbeeb ebbbebbebb**        **f** **f**  **fff**   **f**    **f**        **f**               **s** |
| 601        611        621        631        641         **TAPMQGKRKS ILREPTFRWT SLKHSRSEPQ YFSSAKYAKE GLIRKPIFDN** **eeebeeeeee bbeeeebebb bbeebeeeee bbebbebbee ebbeeebbee**       **ff** **f ssffffsfss s** **f**  **f**   **f** **sfssfssff fssfff** **sff** |
| 651        661        671        681        691         **FRPPPLTPED VGFASGFSAS GTAASARLFS PLHSGTRFDM HKRSPLLRAP** **beeeeeeeee bbbbbbbeee eeebebebbb bbeebbebeb eeebebbebe** **sffffff** **ff sss**                **s**    **f**   **fs**   **fffsf** **sfsf** |
| 701        711        721        731        741         **RFTPSEAHSR IFESVTLPSN RTSAGTSSSG VSNRKRKRKV FSPIRSEPRS** **ebeeeebbbe bbebbbbeee eeeeeeebee eeeeeeeeeb eeebeeeeee** **fsffffsssf s** **fsss**     **f**             **f** **f** **f**       **f**   **ff** |
| 751        761        771        781        791         **PSHSMRTRSG RLSSSELSPL TPPSSVSSSL SISVSPLATS ALNPTFTFPS** **eeeebeeeee ebeeeebeeb eeeeeebebb ebbbbebbbe bbebebebee** **ffffsfffff f**                  **s**           **f ssf**    **s** |
| 801        811        821        831        841         **HSLTQSGESA EKNQRPRKQT SAPAEPFSSS SPTPLFPWFT PGSQTERGRN** **eebeeeeeee eeeeeeeeee eeeeeeeeee eeeebbbbbe eeeeeeeeee**                                      **ss** |
| 851        861        871        881        891         **KDKAPEELSK DRDADKSVEK DKSRERDRER EKENKRESRK EKRKKGSEIQ** **eeebeeeeee eeeeeeeeee eeeeeeeeee eeeeeeeeee eeeeeeeeee**                    **ff**       **f** **f fffff**  **f** **f**  **f** **f** |
| 901        911        921        931        941         **SSSALYPVGR VSKEKVVGED VATSSSAKKA TGRKKSSSHD SGTDITSVTL** **ebebbbebee eeeeeeeeee beeeeebeee eeeeeeeeee ebeebbbbbb**                               **f**   **ff** **f** |
| 951        961        971        981        991         **GDTTAVKTKI LIKKGRGNLE KTNLDLGPTA PSLEKEKTLC LSTPSSSTVK** **eeebebeeee beeeeeeeee eeeeeeeeeb eeeeeeeeee beeeeeeebe**       **f** **f**    **ff** **f** |
| 1001       1011       1021       1031       1041        **HSTSSIGSML AQADKLPMTD KRVASLLKKA KAQLCKIEKS KSLKQTDQPK** **ebbeebbbbb bebeebeeee eebbebbeeb eeebbebeee eeeeeeeeee**     **f**       **f** **ff**    **f ffss** **ss** **fs fffs** **fsff**  **ffff**    **f** |
| 1051       1061       1071       1081       1091        **AQGQESDSSE TSVRGPRIKH VCRRAAVALG RKRAVFPDDM PTLSALPWEE** **eeeeeeeeee beeebeebee bbebbbbebe eeeebbeeeb eebeebeeee**  **f** **fffffff** **fffsffsff ssfssssfsf ffffssfffs ffsffsffff**  26 |
| 1101       1111       1121       1131       1141        **REKILSSMGN DDKSSIAGSE DAEPLAPPIK PIKPVTRNKA PQEPPVKKGR** **eeebbeeeee eeebebeebe ebeeeeeeee ebeeeeeeee eeeeeeeeee** **fff** **sfffff fffsf** **ffsf** **sff** **fff** **f f** **ff** **ff** **f**   **fffffffff** |
| 1151       1161       1171       1181       1191        **RSRRCGQCPG CQVPEDCGVC TNCLDKPKFG GRNIKKQCCK MRKCQNLQWM** **eeeeeeebee eeeeeeeeee eeeeeeeeee eeebeeebbe eeebeebeeb** **fffffffs** **f ffffffff** **f ffffffffff fffsfffssf** **ff** **ffsffs** |
| 1201       1211       1221       1231       1241        **PSKAYLQKQA KAVKKKEKKS KTSEKKDSKE SSVVKNVVDS SQKPTPSARE** **ebeeebeeeb eeeeeeeeee eeeeeeeeee eeeeeeeeee eeeeeeeeee** **fsf**  **sfff**  **f**  **f** **fff**   **f**   **f**                   **f** |
| 1251       1261       1271       1281       1291        **DPAPKKSSSE PPPRKPVEEK SEEGNVSAPG PESKQATTPA SRKSSKQVSQ** **eeeeeeeeee eeeeeeeeee eeeeeeeeee eeeeeeeeee eeeeeeeeee**            **f**                                     **ff** |
| 1301       1311       1321       1331       1341        **PALVIPPQPP TTGPPRKEVP KTTPSEPKKK QPPPPESGPE QSKQKKVAPR** **eeeeeeeeee eeeeeeeebe eeeeeeeeee eeeeeeeeee eeeeeeeeee**                  **f**         **f** **fff** |
| 1351       1361       1371       1381       1391        **PSIPVKQKPK EKEKPPPVNK QENAGTLNIL STLSNGNSSK QKIPADGVHR** **eeeeeeeeee eeeeeeeeee eeeeeebebb bebeeeeeee eeeeeeebbb**          **f**   **f**        **f**       **s**    **f**      **f**    **ffsss** |
| 1401       1411       1421       1431       1441        **IRVDFKEDCE AENVWEMGGL GILTSVPITP RVVCFLCASS GHVEFVYCQV** **bebebeeebe beebbebbbb bbbbbbbbbb ebbbbbbbee eebebbbbeb** **sfsfsf** **f**       **s**  **s**      **ss** **s** **s**  **fsss** **sssff f**  **fss** **sfs** |
| 1451       1461       1471       1481       1491        **CCEPFHKFCL EENERPLEDQ LENWCCRRCK FCHVCGRQHQ ATKQLLECNK** **ebeebeebbb eeeeeebeee beebbbeebe bbebbeeeee ebeebeebee** **fsffsf** **sss ff** **fff** **f** **f** **ffsssffsf ssf** **sffff**    **ff** **ffs** **f** |
| 1501       1511       1521       1531       1541        **CRNSYHPECL GPNYPTKPTK KKKVWICTKC VRCKSCGSTT PGKGWDAQWS** **beeeeeeebe eeeeeeeeee eeebebbbee bebeeeeeee eeeeeebebe**  **fffffffsf fff** **ffffff ff**  **fs** **sff s** **sffff** **ff fff**  **fsf** **f** |
| 1551       1561       1571       1581       1591        **HDFSLCHDCA KLFAKGNFCP LCDKCYDDDD YESKMMQCGK CDRWVHSKCE** **eebebbeebb ebbeeeebbe ebeebeeeee eebebeebee beeebebebe** **ffsf** **sffss fs** **ffff** **sf fsffsfffff ffsfsffs** **f** **f** **f** **f** **fsf** |
| 1601       1611       1621       1631       1641        **NLSGTEDEMY EILSNLPESV AYTCVNCTER HPAEWRLALE KELQISLKQV** **eeeeeeeebb ebbeebeeeb bbbbbebeee eeeebebebe eebeeebeeb**  **ff**    **fss f** **sf** **sfff**  **ssss** **fsf**   **ffffsf** **fs**   **f** **f** **f** |
| 1651       1661       1671       1681       1691        **LTALLNSRTT SHLLRYRQAA KPPDLNPETE ESIPSRSSPE GPDPPVLTEV** **beebbeeebb eebbebeeee eeeeeeeeee eebeeeeeee eeeeebbeeb**  **ffssfffs**  **ffssf** **ff**   **ffffffffff ff** **fffffff fffff** **sffs** |
| 1701       1711       1721       1731       1741        **SKQDDQQPLD LEGVKRKMDQ GNYTSVLEFS DDIVKIIQAA INSDGGQPEI** **eeeeeeeeee eeebeeeeee eebbbbbebb eebbebbbbb beeeeeeeeb**   **f**      **f ff**  **f** **ff**     **s** **ss**   **s ff**     **s** **s** **f** **fff** **ff** |
| 1751       1761       1771       1781       1791        **KKANSMVKSF FIRQMERVFP WFSVKKSRFW EPNKVSSNSG MLPNAVLPPS** **eebeeebebb bbeebeebbe bbebeebebe eeeeeeeeee bbbebbbbeb** **ffsfff** **f**   **s** **ffsff** **sf**   **sffsf** **f f**  **ff**  **fff** **ssfssssfs** |
| 1801       1811       1821       1831       1841        **LDHNYAQWQE REENSHTEQP PLMKKIIPAP KPKGPGEPDS PTPLHPPTPP** **beeebbebee eeeeeeeeee ebbeebbeee eeeeeeeeee eeeeeeeeee** **sfffssfsff ff**      **ff**   **ffssfff**  **f**    **f** **f f** |
| 1851       1861       1871       1881       1891        **ILSTDRSRED SPELNPPPGI EDNRQCALCL TYGDDSANDA GRLLYIGQNE** **eeeeeeeeee eeeeeeeeee eeeeebbbbb eeeeeeeeeb bebbbbbeee**         **ff ff**   **fff**    **ffffs** **sss** **ffff**  **f**   **sfsss** **sfff** |
| 1901       1911       1921       1931       1941        **WTHVNCALWS AEVFEDDDGS LKNVHMAVIR GKQLRCEFCQ KPGATVGCCL** **beebbbbbbb bebbeeeeee beebbbbbbe beebebebbe eeeeebbbbb** **sff** **ssssss sfssffffff sffss** **ss** **f sff** **fsf** **s**    **fffs** **ss** |
| 1951       1961       1971       1981       1991        **TSCTSNYHFM CSRAKNCVFL DDKKVYCQRH RDLIKGEVVP ENGFEVFRRV** **eeeeeebebb bbebeebbbb eeeeeebeeb eebbeeebee eebbebbeeb** **ffffffsfss ssf**   **s** **ss ffffffsffs** **f**  **fff** **f**    **ssfs** **ff** |
| 2001       2011       2021       2031       2041        **FVDFEGISLR RKFLNGLEPE NIHMMIGSMT IDCLGILNDL SDCEDKLFPI** **bbbbebbbbe eebbeebeee ebbbbbbbbb bbbbbbbeeb eebeeebbeb**  **ss** **fs**   **f ff** **s** **fsfff**  **s** **ssssss** **ssss** **s** **fs ffsf** **fssf** |
| 2051       2061       2071       2081       2091        **GYQCSRVYWS TTDARKRCVY TCKIVECRPP VVEPDINSTV EHDENRTIAH** **bbebbebebb beebeeebbb ebebbebeee eeeeebeeeb eeeeebbbee** **ssfssfsfss sffsfffsss fsfs** **f**  **f**      **f** **fff**   **f**  **f** **s**  **f** |
| 2101       2111       2121       2131       2141        **SPTSFTESSS KESQNTAEII SPPSPDRPPH SQTSGSCYYH VISKVPRIRT** **beeebeeeee eeeeeeeeee beeeeeeeee eeeeeebebe ebeeeeebeb** **s**                                                 **f** |
| 2151       2161       2171       2181       2191        **PSYSPTQRSP GCRPLPSAGS PTPTTHEIVT VGDPLLSSGL RSIGSRRHST** **eeeeeeeeee eeeeeeeeee eeeeeeebbb bbbebbeebb ebbbbeeeee**        **ff**   **ffffffff**       **ff** **ss s** **sf** **sffs**       **ffff** |
| 2201       2211       2221       2231       2241        **SSLSPQRSKL RIMSPMRTGN TYSRNNVSSV STTGTATDLE SSAKVVDHVL** **eeeeeeeeeb ebbbeeeeee eebeeeeeeb eeeeeeeeee eeeeeeeebe** **ff** |
| 2251       2261       2271       2281       2291        **GPLNSSTSLG QNTSTSSNLQ RTVVTVGNKN SHLDGSSSSE MKQSSASDLV** **eeeeeeeeee eeeeeeeeee ebeeeeeeee eeeeeeeeee eeeeeeeebb** |
| 2301       2311       2321       2331       2341        **SKSSSLKGEK TKVLSSKSSE GSAHNVAYPG IPKLAPQVHN TTSRELNVSK** **eeeeeeeeee eeeeeeeeee eebebbeeee eeeeeeeeee eeeeeeeeee**          **f**  28 |
| 2351       2361       2371       2381       2391        **IGSFAEPSSV SFSSKEALSF PHLHLRGQRN DRDQHTDSTQ SANSSPDEDT** **eeebeeeeee eeeeeeeeeb eebeeeeeee eeeeeeeeee eeeeeeeeee** |
| 2401       2411       2421       2431       2441        **EVKTLKLSGM SNRSSIINEH MGSSSRDRRQ KGKKSCKETF KEKHSSKSFL** **eeeebeeeee eeebeebeee beeeeeeeee eeeeeeeeeb eeeeeeeebe** |
| 2451       2461       2471       2481       2491        **EPGQVTTGEE GNLKPEFMDE VLTPEYMGQR PCNNVSSDKI GDKGLSMPGV** **eeeeeeeeee eeeeeebeee bbeeeebeee bbeeeeeeee eeeeeeeeee** |
| 2501       2511       2521       2531       2541        **PKAPPMQVEG SAKELQAPRK RTVKVTLTPL KMENESQSKN ALKESSPASP** **eeeeeeeeee eeeeeeeeee eebebebeeb eeeeeeeeee eeeeeeeeee**                     **f**       **ff** |
| 2551       2561       2571       2581       2591        **LQIESTSPTE PISASENPGD GPVAQPSPNN TSCQDSQSNN YQNLPVQDRN** **eeeeeeeeee eeeeeeeeee eeeeeeeeee eeeeeeeeee eeebeeeeee** |
| 2601       2611       2621       2631       2641        **LMLPDGPKPQ EDGSFKRRYP RRSARARSNM FFGLTPLYGV RSYGEEDIPF** **bbbeeeeeee eeeeeeeebe eeebebebeb bbbbbbbbbb eeeeeeebeb**        **f**   **f**  **f** **fffsf fffsfsfsfs ssssss** **sss fffffff** **fs** |
| 2651       2661       2671       2681       2691        **YSSSTGKKRG KRSAEGQVDG ADDLSTSDED DLYYYNFTRT VISSGGEERL** **eeeeeeeeee eeeeeeebee beeeeeeeee ebbbbbbbbb ebbeeeeeeb**  **f** **f** **ffff**  **fffffffsff sff** **ffff**   **f** **sssss** **ss f**       **f** |
| 2701       2711       2721       2731       2741        **ASHNLFREEE QCDLPKISQL DGVDDGTESD TSVTATTRKS SQIPKRNGKE** **eeeebbeeee eeebeebeeb eeeeeeeeee eebebbeeeb eeeeeeeeee**      **sf** **f**        **sffs fffff** **ffff** **f**             **ff**  **ff** |
| 2751       2761       2771       2781       2791        **NGTENLKIDR PEDAGEKEHV TKSSVGHKNE PKMDNCHSVS RVKTQGQDSL** **eeeeebeeee eeeeeeeeeb beeeeeeeee eeeeebeeeb ebeeeeeebb** **ff**               **f**           **f**    **f**  **f** **f**      **f**   **ff** |
| 2801       2811       2821       2831       2841        **EAQLSSLESS RRVHTSTPSD KNLLDTYNTE LLKSDSDNNN SDDCGNILPS** **eeebeeeeeb eebeeeeeee eebbeebbee bbeeeeeeee eeebeebbbb**   **f** **fff**    **ff** **f**   **f**   **f**   **f**      **s** **ffffffff fff** **ffssss** |
| 2851       2861       2871       2881       2891        **DIMDFVLKNT PSMQALGESP ESSSSELLNL GEGLGLDSNR EKDMGLFEVF** **ebbbbbbeee eebebeeeee eeeeeebbeb beebeeeeee eeebbbbebb** **fssssssfff ffsf** **fffff ffffffs**  **s**  **f** **fff** **ff** **ff**  **ssfss** |
| 2901       2911       2921       2931       2941        **SQQLPTTEPV DSSVSSSISA EEQFELPLEL PSDLSVLTTR SPTVPSQNPS** **eeebeeeeee eeebbbbbee eeebebeeeb eeebebbbee eeeeeeeeee** **fffs**   **f** **f ff** **s**    **f**  **ff**  **fsfffs fffsfsssff ff** **f**  **ff**  29 |
| 2951       2961       2971       2981       2991        **RLAVISDSGE KRVTITEKSV ASSESDPALL SPGVDPTPEG HMTPDHFIQG** **ebbbbeeeee eebebeeeee eeeeeeebbb eeeeeeeeee ebeeeebbeb**     **s**      **ff**     **f**                         **f** **fff** **s** |
| 3001       3011       3021       3031       3041        **HMDADHISSP PCGSVEQGHG NNQDLTRNSS TPGLQVPVSP TVPIQNQKYV** **bbebeebeee eeeeeeeeee eeeebeeeee eeebebeeee ebeeeeeeee**      **f**  **ff**             **f**   **f**    **ff**    **ffff**       **ff** |
| 3051       3061       3071       3081       3091        **PNSTDSPGPS QISNAAVQTT PPHLKPATEK LIVVNQNMQP LYVLQTLPNG** **eeeeeeeeee ebbeebbeeb eeebeebeee bbbbeeebee bebbeebeee**        **f** **f**       **f**     **f** **f**    **f**  **ssfff** **ff sfs** **ff** **fff** |
| 3101       3111       3121       3131       3141        **VTQKIQLTSS VSSTPSVMET NTSVLGPMGG GLTLTTGLNP SLPTSQSLFP** **beeebebeee eeebeebbee ebebbbbbbe bbbbbeeeee eeeeeeeebe**  **fffsf** **f**   **f**       **f**      **s**         **s**              **f** |
| 3151       3161       3171       3181       3191        **SASKGLLPMS HHQHLHSFPA ATQSSFPPNI SNPPSGLLIG VQPPPDPQLL** **eebeebbebe eeebbbebee beeeeeeeee eeeeebbbbb beeeeeeebb**    **f**       **f**      **s**        **f**           **s** **ss sf**     **f** **s** |
| 3201       3211       3221       3231       3241        **VSESSQRTDL STTVATPSSG LKKRPISRLQ TRKNKKLAPS STPSNIAPSD** **beeeeeeeee eeeeeeeeee beeeebeebe eeeeeeeeee eeeeebbbee**  **ff**   **f**                **fff** **sffs**   **f**  **ff** **fff** |
| 3251       3261       3271       3281       3291        **VVSNMTLINF TPSQLPNHPS LLDLGSLNTS SHRTVPNIIK RSKSSIMYFE** **bbbbbbbbbb beeebeeeee bbebbbbeee eeeebeebbe ebeeebbbbe**    **sss**  **s**     **f**   **f**     **fs**        **ff**  **ffssf f** **f**    **ssf** |
| 3301       3311       3321       3331       3341        **PAPLLPQSVG GTAATAAGTS TISQDTSHLT SGSVSGLASS SSVLNVVSMQ** **ebeebeeeee eebbebeeee ebbeeeeebe ebebebbbee eebbbbbbbe**    **f** **f**                                      **f**  **sss** |
| 3351       3361       3371       3381       3391        **TTTTPTSSAS VPGHVTLTNP RLLGTPDIGS ISNLLIKASQ QSLGIQDQPV** **beeeeeeeee eeeebbbebe ebbbebeebb bbebbbebbe eeeebeeeee**                  **s**                  **ss** **f**    **f** **f** |
| 3401       3411       3421       3431       3441        **ALPPSSGMFP QLGTSQTPST AAITAASSIC VLPSTQTTGI TAASPSGEAD** **ebbeeeeebb eeeeeeeeee bbbbbbebbb bbeeeeebeb ebeeeeeeee**                                    **f** |
| 3451       3461       3471       3481       3491        **EHYQLQHVNQ LLASKTGIHS SQRDLDSASG PQVSNFTQTV DAPNSMGLEQ** **eeeebeebee bbeeeeeeee eeeeeeeeee eeeeebeebb eeeeeeeeee** |
| 3501       3511       3521       3531       3541        **NKALSSAVQA SPTSPGGSPS SPSSGQRSAS PSVPGPTKPK PKTKRFQLPL** **eeeeeeebeb eeeeeeebeb eeeeeeeeee eeeeeeeeee eebeebeeee**                       **f**                   **f** **f** **ff** |
| 3551       3561       3571       3581       3591        **DKGNGKKHKV SHLRTSSSEA HIPDQETTSL TSGTGTPGAE AEQQDTASVE** **eeeeeeeeee eebeeeeeee ebeeeeeeee eeeeeeeeee eeeeeeeeee**      **ffff**  30 |
| 3601       3611       3621       3631       3641        **QSSQKECGQP AGQVAVLPEV QVTQNPANEQ ESAEPKTVEE EESNFSSPLM** **eeeeeeeeee eeeeeeeeee eeeeeeeeee eeeeeeeeee eeeebebebb**                                                  **f** **f** |
| 3651       3661       3671       3681       3691        **LWLQQEQKRK ESITEKKPKK GLVFEISSDD GFQICAESIE DAWKSLTDKV** **bbbeeeeeee eeeeeeeeee ebbbebbeee bbebbbebbe ebbeebeeeb**      **f**              **f** **s** **sfs** **fff ssfs**  **f**  **f** **ssffsfffs** |
| 3701       3711       3721       3731       3741        **QEARSNARLK QLSFAGVNGL RMLGILHDAV VFLIEQLSGA KHCRNYKFRF** **eebebeeebe ebbbebbbbb bbbbbbbebb bbbbeebeeb eebeebebeb** **ffsfsfffsf f** **ssfss** **s**   **sss**  **sfss** **ss** **ffs** **fs** **fs** **fs** **sfs** |
| 3751       3761       3771       3781       3791        **HKPEEANEPP LNPHGSARAE VHLRKSAFDM FNFLASKHRQ PPEYNPNDEE** **eeeeeeeeee eeeeeebebe bebeebbbeb bebbbeeeee eeeeeeeeee** **f** **f** **f**    **f** **fffffsfsf** **f** **f** **s** **sfs sfsssfffff ff** **f** **f**  **ff** |
| 3801       3811       3821       3831       3841        **EEEVQLKSAR RATSMDLPMP MRFRHLKKTS KEAVGVYRSP IHGRGLFCKR** **eeeeebeebe ebebeebebe bbbeebeeeb eebbebbebb bebebbbbee** **fffffsffsf f** **fsffsf**    **ssffs**  **fs ff** **sfs** **fs**  **sfsfssssff** |
| 3851       3861       3871       3881       3891        **NIDAGEMVIE YAGNVIRSIQ TDKREKYYDS KGIGCYMFRI DDSEVVDATM** **ebebbebbbe bbbebbbbbb eeeeeeeeee eebbbbbbbb eeeebbbbbb** **fsfssfsssf sssfssss** **s fffffffff**  **ffsss** **ssss ff** **fsssss** |
| 3901       3911       3921       3931       3941        **HGNAARFINH SCEPNCYSRV INIDGQKHIV IFAMRKIYRG EELTYDYKFP** **bbbbbebbeb ebeeebbbeb bebeeeebbb bbbbbebeee eebebebebe** **sssssfssfs f** **fffs** **sfs**   **ffffsss sss** **sfs** **ff ffsfsfsfsf** |
| 3951       3961       3971        **IEDASNKLPC NCGAKKCRKF LN** **beebeeebeb eeeeeebeee ee** **sffs** **ffs** **s ffffffsfff ff** |


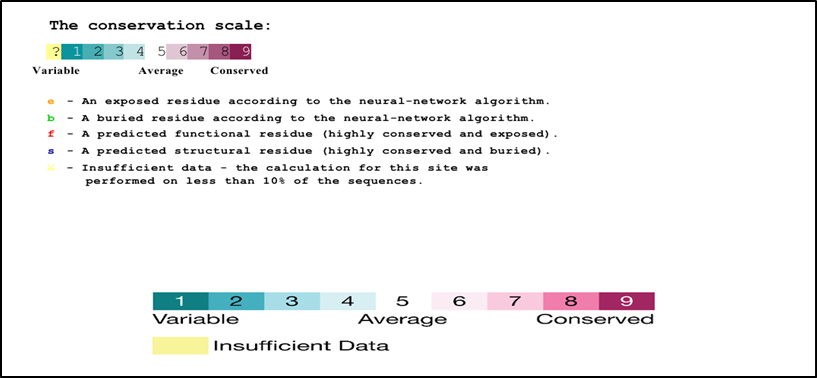


**Figure S2**. Evolutionary conservation analysis performed for *MLL1* protein sequence 1 to 3969 aa using ConSurf.Amino acids are shaded according to their conservation ratings and levels. A grade of 1 represents fast-evolving (variable) sites and is marked in turquoise; a grade of 5 denotes sites evolving at a standard rate, shown in white; while a grade of 9 points to (evolutionarily conserved) sites that evolve slowly, represented in maroon. When a particular position covers a range of 4 or less color grades, its score is viewed as unreliable. These positions are highlighted in light yellow in the visual display.

| **Table S3**. RegulomeDB variant classification scheme | |
| --- | --- |
| **Description** | **Category** |
| Likely to affect binding and linked to the expression of a gene target | |
| eQTL+ TF binding + matched TF motif + matched DNase footprint +DNase peak | 1a |
| eQTL+ TF binding + any motif + DNase footprint +DNase peak | 1b |
| eQTL+ TF binding + matched TF motif +DNase peak | 1c |
| eQTL+ TF binding + any motif + DNase footprint +DNase peak | 1d |
| eQTL+ TF binding + matched TF motif | 1e |
| eQTL+ TF binding / DNase peak | 1f |
| Likely to affect the binding | |
| TF binding + matched TF motif + matched DNase footprint +DNase peak | 2a |
| TF binding + any motif +DNase footprint +DNase peak | 2b |
| TF binding + matched TF motif +DNase peak | 2c |
| Less likely to affect the binding | |
| TF binding + any motif +DNase peak | 3a |
| TF binding + matched TF motif | 3b |
| Minimal binding evidence | |
| TF binding +DNase peak | 4 |
| TF binding or DNase peak | 5 |
| Motif hit | 6 |

**Table S4.** The effect of the nsSNPs on *MLL1* protein structure using Project HOPE

| **SNPID** | **AA change** | **Domain** | **AA proparties** |
| --- | --- | --- | --- |
| rs1085307947 | C1072F | Zinc finger, CXXC-type | - The wild-type and mutant amino acids differ in size. - The mutant residue is bigger, this might lead to bumps. |
| rs1057518074 | C1155Y | Zinc finger, CXXC-type | - The wild-type and mutant amino acids differ in size. - The mutant residue is bigger, this might lead to bumps. - The hydrophobicity of the wild-type and mutant residue differs. - Hydrophobic interactions, either in the core of the protein or on the surface, will be lost. |
| rs1131691503 | C1158Y | Zinc finger, CXXC-type | - The wild-type and mutant amino acids differ in size. - The mutant residue is bigger, this might lead to bumps. - The hydrophobicity of the wild-type and mutant residue differs. - Hydrophobic interactions, either in the core of the protein or on the surface, will be lost. |
| rs1555038115 | G1180V | Zinc finger, CXXC-type | - The wild-type and mutant amino acids differ in size. - The mutant residue is bigger, this might lead to bumps. - The torsion angles for this residue are unusual. only glycine is flexible enough to make these torsion angles, mutation into another residue will force the local backbone into an incorrect conformation and will disturb the local structure. |
| rs1950071303 | G1181C | Zinc finger, CXXC-type | - The wild-type and mutant amino acids differ in size. - The mutant residue is bigger, this might lead to bumps. - The torsion angles for this residue are unusual. only glycine is flexible enough to make these torsion angles, mutation into another residue will force the local backbone into an incorrect conformation and will disturb the local structure. |
| rs886041875 | C1189R | Zinc finger, CXXC-type | - There is a difference in charge between the wild-type and mutant amino acid. - The mutation introduces a charge, this can cause repulsion of ligands or other residues with the same charge. - The wild-type and mutant amino acids differ in size. - The mutant residue is bigger, this might lead to bumps. - The hydrophobicity of the wild-type and mutant residue differs. - Hydrophobic interactions, either in the core of the protein or on the surface, will be lost. |
| rs1555038125 | C1189Y | Zinc finger, CXXC-type | - The wild-type and mutant amino acids differ in size. - The mutant residue is bigger, this might lead to bumps. - The hydrophobicity of the wild-type and mutant residue differs. - Hydrophobic interactions, either in the core of the protein or on the surface, will be lost. |
| rs1950106455 | C1194Y | Zinc finger, CXXC-type | - The wild-type and mutant amino acids differ in size. - The mutant residue is bigger, this might lead to bumps. - The hydrophobicity of the wild-type and mutant residue differs. - Hydrophobic interactions, either in the core of the protein or on the surface, will be lost. |
| rs863224895 | C1448R | PHD1-3 | - There is a difference in charge between the wild-type and mutant amino acid. - The mutation introduces a charge, this can cause repulsion of ligands or other residues with the same charge. - The wild-type and mutant amino acids differ in size. - The mutant residue is bigger, this might lead to bumps. - The hydrophobicity of the wild-type and mutant residue differs. - Hydrophobic interactions, either in the core of the protein or on the surface, will be lost. |
| rs1085307857 | C1448Y | PHD1-3 | - The wild-type and mutant amino acids differ in size. - The mutant residue is bigger, this might lead to bumps. - The hydrophobicity of the wild-type and mutant residue differs. - Hydrophobic interactions, either in the core of the protein or on the surface, will be lost. |
| rs1555042404 | C1588F | PHD1-3 | - The wild-type and mutant amino acids differ in size. - The mutant residue is bigger, this might lead to bumps. |
| rs782594163 | W1635C | Bromodomain | - The wild-type and mutant amino acids differ in size. - The mutant residue is smaller, this might lead to loss of interactions. |
| rs373435126 | R1658W | Bromodomain | - There is a difference in charge between the wild-type and mutant amino acid. - The charge of the wild-type residue will be lost, this can cause loss of interactions with other molecules or residues. - The wild-type and mutant amino acids differ in size. - The mutant residue is bigger, this might lead to bumps. - The hydrophobicity of the wild-type and mutant residue differs. - The mutation introduces a more hydrophobic residue at this position. This can result in loss of hydrogen bonds and/or disturb correct folding. |
| rs781944403 | R1763P | Bromodomain | - There is a difference in charge between the wild-type and mutant amino acid. - The charge of the wild-type residue will be lost, this can cause loss of interactions with other molecules or residues. - The wild-type and mutant amino acids differ in size. - The mutant residue is smaller, this might lead to loss of interactions. - The hydrophobicity of the wild-type and mutant residue differs. - The mutation introduces a more hydrophobic residue at this position. This can result in loss of hydrogen bonds and/or disturb correct folding. |
| rs1555044535 | V1924M | Extended PHD 4 | - The wild-type and mutant amino acids differ in size. - The mutant residue is bigger than the wild-type residue. - The wild-type residue was buried in the core of the protein. The mutant residue is bigger and probably will not fit. |
| rs1950444447 | G1943E | Extended PHD 4 | - There is a difference in charge between the wild-type and mutant amino acid. - The mutant residue introduces a charge in a buried residue which can lead to protein folding problems. - The wild-type and mutant amino acids differ in size. - The mutant residue is bigger than the wild-type residue. - The wild-type residue was buried in the core of the protein. The mutant residue is bigger and probably will not fit. - The torsion angles for this residue are unusual. only glycine is flexible enough to make these torsion angles, mutation into another residue will force the local backbone into an incorrect conformation and will disturb the local structure. |
| rs782768278 | R2067H | F/Y-rich N-terminus | - There is a difference in charge between the wild-type and mutant amino acid. - The charge of the wild-type residue will be lost, this can cause loss of interactions with other molecules or residues. - The wild-type and mutant amino acids differ in size. - The mutant residue is smaller, this might lead to loss of interactions. |
| rs1555050212 | R3704Q | FYR C-terminal | - There is a difference in charge between the wild-type and mutant amino acid. - The charge of the wild-type residue will be lost, this can cause loss of interactions with other molecules or residues. - The wild-type and mutant amino acids differ in size. - The mutant residue is smaller, this might lead to loss of interactions. |
| rs782658611 | C3743Y | FYR C-terminal | - The wild-type and mutant amino acids differ in size. - The mutant residue is bigger, this might lead to bumps. - The hydrophobicity of the wild-type and mutant residue differs. - Hydrophobic interactions, either in the core of the protein or on the surface, will be lost. |
| rs749354451 | F3748C | FYR C-terminal | - There is a difference in charge between the wild-type and mutant amino acid. - The charge of the wild-type residue will be lost, this can cause loss of interactions with other molecules or residues. - The wild-type and mutant amino acids differ in size. - The mutant residue is smaller, this might lead to loss of interactions. - The hydrophobicity of the wild-type and mutant residue differs. - The mutation introduces a more hydrophobic residue at this position. This can result in loss of hydrogen bonds and/or disturb correct folding. |
| rs782366377 | R3749C | FY-rich, C-terminal | - There is a difference in charge between the wild-type and mutant amino acid. - The charge of the wild-type residue will be lost, this can cause loss of interactions with other molecules or residues. - The wild-type and mutant amino acids differ in size. - The mutant residue is smaller, this might lead to loss of interactions. |
| rs781821970 | D2724G |  | - The wild-type and mutant amino acids differ in size. - The mutant residue is smaller, this might lead to loss of interactions. |
| rs1555048205 | L3393P |  | - The wild-type and mutant amino acids differ in size. - The mutant residue is smaller, this might lead to loss of interactions. |
| rs782600332 | E3860D | SET domain | - The wild-type and mutant amino acids differ in size. - The mutant residue is smaller, this might lead to loss of interactions. |
| rs1591311290 | G3863S | SET domain | - The wild-type and mutant amino acids differ in size. - The mutant residue is bigger, this might lead to bumps. - The torsion angles for this residue are unusual. only glycine is flexible enough to make these torsion angles, mutation into another residue will force the local backbone into an incorrect conformation and will disturb the local structure. |
| rs781980190 | E3875V | SET domain | - There is a difference in charge between the wild-type and mutant amino acid. - The charge of the wild-type residue will be lost, this can cause loss of interactions with other molecules or residues. - The wild-type and mutant amino acids differ in size. - The mutant residue is smaller, this might lead to loss of interactions. - The hydrophobicity of the wild-type and mutant residue differs. - The mutation introduces a more hydrophobic residue at this position. This can result in loss of hydrogen bonds and/or disturb correct folding. |
| rs1555053677 | R3889Q | SET domain | - There is a difference in charge between the wild-type and mutant amino acid. - The charge of the wild-type residue will be lost, this can cause loss of interactions with other molecules or residues. - The wild-type and mutant amino acids differ in size. - The mutant residue is smaller, this might lead to loss of interactions. |
